# Supplementary material for: Hand Grip Strength Relative to Waist Circumference as a Means to Identify Men and Women Possessing Intact Mobility in a Cohort of Older Adults with Type 2 Diabetes
Source: Biomedicines. 2023 Jan 26;11(2):352. doi: 10.3390/biomedicines11020352 (PMC9953481; doi:10.3390/biomedicines11020352)
Supplement: Supplementary file 1 [file biomedicines-11-00352-s001.zip › biomedicines-2154221-supplementary.pdf]

Supplementary File for "Hand Grip Strength Relative to Waist Circumference as a Means to Identify Men and Women Possessing Intact Mobility in a Cohort of Older Adults with Type 2 Diabetes"

Table S1. Univariate independent t-tests performed between each HGS index and patients passing cut-off points of all mobility tests (mobility intact). (**a**) Independent Samples Test men. (**b**) Independent Samples Test women.

(**a**)

HGS index	t-test for Equality of Means						
	t	df	Sig. (2-tailed)	Mean Difference	Std. Error Difference	95% Confidence Interval of the Difference	
						Lower	Upper
HGS	-2.813	48	0.007	-5.0217	1.7854	-8.6115	-1.4318
HGS/BMI	-3.032	48	0.004	-0.25200	0.08313	-0.41914	-0.08486
HGS/FM	-2.762	48	0.008	-0.41550	0.15045	-0.71801	-0.11299
HGS/SMM	-1.196	48	0.238	-0.09750	0.08152	-0.26141	0.06641
HGS/PBF	-2.886	48	0.006	-0.29533	0.10234	-0.50110	-0.08957
HGS/WC	-3.548	48	0.001	-0.07333	0.02067	-0.11489	-0.03177
HGS/BW	-3.282	48	0.002	-0.08400	0.02560	-0.13546	-0.03254
HGS/H	-2.963	48	0.005	-2.92117	0.98573	-4.90311	-0.93922

(**b**)

HGS index	t-test for Equality of Means						
	t	df	Sig. (2-tailed)	Mean Difference	Std. Error Difference	95% Confidence Interval of the Difference	
						Lower	Upper
HGS	-1.789	70	0.078	-2.0172	1.1277	-4.2663	0.2318
HGS/BMI	-3.092	70	0.003	-0.14803	0.04787	-0.24351	-0.05255
HGS/FM	-2.617	70	0.011	-0.16363	0.06252	-0.28831	-0.03895
HGS/SMM	-1.942	70	0.056	-0.13161	0.06777	-0.26678	0.00357
HGS/PBF	-2.476	70	0.016	-0.08074	0.03261	-0.14577	-0.01570
HGS/WC	-3.255	70	0.002	-0.03808	0.01170	-0.06141	-0.01475
HGS/BW	-3.094	70	0.003	-0.05477	0.01770	-0.09007	-0.01946
HGS/H	-1.647	70	0.104	-1.11711	0.67817	-2.46967	0.23545

Abbreviations: BMI, Body Mass Index; FM, fat mass; H, height; HGS, hand-grip strength; PBF, percent body fat; SMM, skeletal muscle mass; WC, waist circumference.

Table S2. Pearson Chi-Square tests of possible confounders and passing all three mobility tests.

Confounder	Value	Df	Asymp. Sig. (2-sided)
**Men**			
Hypertension	2.037	1	0.153
Diabetes complications [1]	0.055	1	0.815
Physical activity [2]	5.333	1	0.021 *
Body Mass Index (≥30)	2.391	1	0.122
Vitamin D (<25 ng/ml)	0.033	1	0.856
Polypharmacy (≥8 drugs)	0.306	1	0.580
**Women**			
Hypertension	0.832	1	0.362
Diabetes complications [1]	0.795	1	0.373
Physical activity [2]	6.926	1	0.008 *
Body Mass Index (≥30)	4.281	1	0.039 *
Vitamin D (<25 ng/ml)	2.053	1	0.152
Polypharmacy (≥8 drugs)	1.475	1	0.225

* $p < 0.05$; [1] diagnosis of neuropathy, retinopathy, chronic kidney disease, ischemic heart disease; [2] Performing ≤ 2 days a week of any leisure aerobic physical activity.
